# Class 1 integron-borne cassettes harboring *bla*_CARB-2_ gene in multidrug-resistant and virulent *Salmonella* Typhimurium ST19 strains recovered from clinical human stool samples, United States

**DOI:** 10.1371/journal.pone.0240978

**Published:** 2020-10-30

**Authors:** Daniel F. M. Monte, Fábio P. Sellera, Ralf Lopes, Shivaramu Keelara, Mariza Landgraf, Shermalyn Greene, Paula J. Fedorka-Cray, Siddhartha Thakur

**Affiliations:** 1 Department of Food and Experimental Nutrition, Food Research Center, Faculty of Pharmaceutical Sciences, University of São Paulo, São Paulo, Brazil; 2 Department of Population Health and Pathobiology, North Carolina State University, College of Veterinary Medicine, Raleigh, North Carolina, United States of America; 3 Department of Internal Medicine, School of Veterinary Medicine and Animal Science, University of São Paulo, São Paulo, Brazil; 4 Department of Clinical Analyses, Toxicology and Food Science, School of Pharmaceutical Sciences of Ribeirão Preto, University of São Paulo, Ribeirão Preto, Brazil; 5 Department of Health and Human Services, Molecular Diagnostic and Epidemiology Laboratory Unit at State Laboratory of Public Health, Division of Public Health, Raleigh, North Carolina, United States of America; 6 Comparative Medicine Institute, North Carolina State University, Raleigh, North Carolina, United States of America; Nitte University, INDIA

## Abstract

International lineages, such as *Salmonella* Typhimurium sequence type (ST) 19, are most often associated with foodborne diseases and deaths in humans. In this study, we compared the whole-genome sequences of five *S*. Typhimurium strains belonging to ST19 recovered from clinical human stool samples in North Carolina, United States. Overall, *S*. Typhimurium strains displayed multidrug-resistant profile, being resistance to critically and highly important antimicrobials including ampicillin, ticarcillin/clavulanic acid, streptomycin and sulfisoxazole, chloramphenicol, tetracycline, respectively. Interestingly, all *S*. Typhimurium strains carried class 1 integron (*intl1*) and we were able to describe two genomic regions surrounding *bla*_CARB-2_ gene, size 4,062 bp and 4,422 bp for *S*. Typhimurium strains (HS5344, HS5437, and HS5478) and (HS5302 and HS5368), respectively. Genomic analysis for antimicrobial resistome confirmed the presence of clinically important genes, including *bla*_CARB-2_, *aac(6’)-Iaa*, *aadA2b*, *sul1*, *tetG*, *floR*, and biocide resistance genes (*qacEΔ1*). *S*. Typhimurium strains harbored IncFIB plasmid containing *spvRABCD* operon, as well as *rck* and *pef* virulence genes, which constitute an important apparatus for spreading the virulence plasmid. In addition, we identified several virulence genes, chromosomally located, while the phylogenetic analysis revealed clonal relatedness among these strains with *S*. *enterica* isolated from human and non-human sources obtained in European and Asian countries. Our results provide new insights into this unusual class 1 integron in virulent *S*. Typhimurium strains that harbors a pool of genes acting as potential hotspots for horizontal gene transfer providing readily adaptation to new surrounds, as well as being crucially required for virulence *in vivo*. Therefore, continuous genomic surveillance is an important tool for safeguarding human health.

## Introduction

Non-typhoidal *Salmonella* (NTS) is one of the most important foodborne pathogens with unprecedented impact on global health [1]. Among NTS, *Salmonella enterica* subsp. *enterica* serovar Typhimurium represents a major threat, since its worldwide spread has been associated with a broad host range, which includes mostly humans and food-related sources [1, 2]. Besides that, the emergence of multidrug-resistant (MDR) *S*. *enterica* is another crucial aspect for food-related outbreaks globally, limiting our therapeutic options [3].

In addition to the high global burden of salmonellosis, extended-spectrum β-lactamase (ESBL)-producing *S*. *enterica* strains have been recognized as high-priority bacteria causing serious public health issue (https://www.who.int/news-room/detail/27-02-2017-who-publishes-list-of-bacteria-for-which-new-antibiotics-are-urgently-needed). Aside from this, the emergence of mobile genetic elements (MGEs), for instance, class 1 integrons play an essential role in the global spread of antimicrobial resistance [4, 5]. Another aspect to be considered is the wide range of virulence package that is typically associated with *Salmonella* Pathogenicity Islands (SPI), contributing to the infection process among diverse hosts [6–8]. In this context, while the surveys with genomic approach have helped in the development of mitigation strategies and clinical management, continuous active surveillance is urgently required.

Here, we describe the genomic characteristics of five MDR and virulent *S*. Typhimurium strains carrying the *bla*_CARB-2_ gene recovered from clinical human stool samples in North Carolina, United States.

## Materials and methods

### Ethics approval and consent to participate

The human patients from whom *Salmonella* were recovered were completely anonymous and even after all the analysis and tests, the human sample remained anonymous. As such, the NC State IRB (FWA: 00003429) indicated the study research did not need IRB approval because it does not meet the definition for human subjects research.

### Bacterial strains and antimicrobial susceptibility testing

We conducted a genomic investigation on five clinical *S*. Typhimurium strains collected in 2014 in North Carolina, United States. The strains were subjected to phenotypic characterization using the microdilution panel susceptibility approach on Gram-negative Sensititre plates (CMV3AGNF and GNX2F, Trek Diagnostic Systems, OH, USA) following the interpretative criteria of Clinical and Laboratory Standards Institute [9, 10]. The MDR profile was defined as resistant to three or more classes of antimicrobials [11]. All *S*. Typhimurium strains underwent molecular screening for class 1 integron by PCR [12, 13] and were subsequently characterized by whole-genome sequencing (WGS) according to Pornsukarom et al. [14].

### Whole-genome sequencing and phylogenetic analysis

Libraries were prepared using the Nextera XT DNA sample preparation kit (Illumina, San Diego, CA), which were multiplexed and sequenced on MiSeq platform (Illumina, San Diego, CA, USA) at a paired-end read (300 bp). Resulted raw sequence reads underwent a strict quality control, as well as we obtained the draft genomes by using default settings in CLC workbench 10.1.1 (Qiagen) as per Monte et al. [15]. The sequencing data were deposited in NCBI (PRJNA613764). For each strain, we uploaded the sequences into Center for Genomic Epidemiology (http://genomicepidemiology.org/) to detect multilocus sequence typing (MLST), resistome, plasmid incompatibility groups and *Salmonella* Pathogenicity Islands.

Virulome analyzes were performed by using default settings available in VFanalyzer [16]. Additionally, the genetic context of *bla*_CARB-2_ and presence of virulence genes were investigated using BLASTn analysis against the non-redundant (NR) database and manually curated using Geneious v. 11.1.5 (Biomatters Ltd., Auckland, New Zealand).

For phylogenetic purpose, we reconstructed a maximum likelihood phylogenetic tree based on single nucleotide polymorphism (SNP) using default settings of CSI Phylogeny version 1.4 [17]. SNP tree was reconstructed with five genomes of *S*. Typhimurium from this study in addition to thirteen genomes retrieved from GenBank database. Additional genomes of *S*. *enterica* strains were chosen from different sources (human, camel, food, poultry, ovine, river, and dog) and countries, including USA (SAMN10863500 and SAMEA6514930), France (SAMN07734943), Scotland (SAMEA773504 and SAMEA773551), Denmark (SAMEA4349586), Ireland (SAMEA4825483), Switzerland (SAMN08936646), Germany (SAMEA6058372), Chile (SAMN14336901), China (SAMN09759463 and SAMN02844307), and Ethiopia (SAMN03577126).

## Results

### Antimicrobial susceptibility testing and class 1 integron detection

All the five strains were classified as MDR, displaying resistance to critically important antimicrobials including ampicillin (100%), ticarcillin/clavulanic acid (100%), and streptomycin (60%), as well as to highly important antimicrobials comprising sulfisoxazole (100%), chloramphenicol (100%), and tetracycline (60%) (Table 1). Moreover, intermediate resistance to doxycycline was detected in three strains (HS5344, HS5437 and HS5478), and in a single strain (HS5437) to ceftazidime. In addition, we confirmed the presence of class 1 integron in all *S*. Typhimurium strains.

**Table 1:** Phenotypic and genomic features of *Salmonella* Typhimurium ST19 strains isolated from clinical human samples in United States.

Strain ID	Serotype	Source	R-type (MIC)*	Resistance genotype	Plasmids	ST	Accession number
HS5302	Typhimurium (O5-)	Stool	FIS-AMP-TIM2	*bla*_CARB-2_, *aac(6’)-Iaa*, *sul1*	IncFIB(S), IncFII(S)	19	JAATJP000000000
HS5344	Typhimurium (O5-)	Stool	CHL-TET-FIS-AMP-STR-TIM2	*bla*_CARB-2_, *aac(6’)-Iaa*, *aadA2b*, *sul1*, *tet(G)*, *floR*	IncFIB(S), IncFII(S)	19	JAATGY000000000
HS5368	Typhimurium (O5-)	Stool	FIS-AMP-TIM2	*bla*_CARB-2_, *aac(6’)-Iaa*, *sul1*	IncFIB(S), IncFII(S)	19	JAATJO000000000
HS5437	Typhimurium (O5-)	Stool	CHL-TET-FIS-AMP-STR-TIM2	*bla*_CARB-2_, *aac(6’)-Iaa*, *aadA2b*, *sul1*, *tet(G)*, *floR*	IncFIB(S), IncFII(S)	19	JAATGZ000000000
HS5478	Typhimurium	Stool	CHL-TET-FIS-AMP-STR-TIM2	*bla*_CARB-2_, *aac(6’)-Iaa*, *aadA2b*, *aph(3’)-Ia*, *sul1*, *tet(G)*, *floR*	IncFIB(S), IncFII(S)	19	JAATHA000000000

*FIS, sulfisoxazole; AMP, ampicillin; TIM2, ticarcillin/clavulanic acid constant 2; CHL, chloramphenicol; TET, tetracycline; STR, streptomycin.

### Whole-genome sequencing and phylogenetic analysis

**Fig 1:**
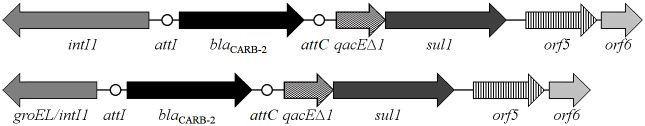
Schematic representation of the genetic context surrounding *bla*_CARB-2_ genes in *Salmonella* Typhimurium ST19 strains isolated from clinical human samples in United States.

Genomic analysis revealed that all five *S*. Typhimurium strains belonged to the international sequence type (ST) ST19, while antimicrobial resistome confirmed the presence of critically important genes, such as carbenicillinase [*bla*_CARB-2_], aminoglycosides [*aac(6’)-Iaa* and *aadA2b*], sulfonamide [*sul1*], tetracycline [*tetG*], and florfenicol [*floR*]. The IncFIB(S) and IncFII(S) plasmid incompatibility groups were detected in all strains. We were also able to describe two schematic representations of the genetic context surrounding *bla*_CARB-2_ gene. First, three *S*. Typhimurium strains (HS5344, HS5437, and HS5478) analyzed in this study, shared a genomic environment with 4,062 bp in size composed by *groEL*/*intI1*-*bla*_CARB-2_-*qacEΔ1*-*sul1*-*orf5* (acetyltransferase)-*orf6* (hypothetical protein). Second, the remaining *S*. Typhimurium strains (HS5302 and HS5368) presented a genomic content slightly different with a 4,422 bp region composed by *intI1*-*bla*_CARB-2_-*qacEΔ1*-*sul1*-*orf5* (acetyltransferase)-*orf6* (hypothetical protein) (Fig 1). Additionally, the *sul1*, *bla*_CARB-2_, *tetG*, *floR*, and *aadA2b* resistance genes were harbored by a partial sequence of a complex class 1 integron (In104) from HS5344, HS5437, and HS5478. This sequence included duplications of parts of the integron conserved segments (CS), specifically, part of the *intI1* gene from the 5’-CS and part of the 3’-CS (*qacEΔ1* and partial *sul1* genes). Consequently, the structure had two *attI1* sites, into which the *aadA2b* gene cassette was inserted in one and the *bla*_CARB-2_ cassette in the other. The *floR* and *tetG* genes were identified between the two integron-derived regions. In HS5302 and HS5368, only the region containing the *intI1*-*bla*_CARB-2_-*qacEΔ1*-*sul1*-*orf5*-*orf6* array was detected. Furthermore, while *aac(6’)-Iaa* was found at a site distant from the other resistance genes on the chromosome of all *S*. Typhimurium strains in this study, *aph(3’)-Ia* was identified in a partial transposon sequence from HS5478.

Virulome analysis revealed presence of several *Salmonella* Pathogenicity Island (SPI-1, SPI-2, SPI-3, SPI-4, SPI-5, SPI-13, SPI-14, and Centisome 63 Pathogenicity Island) as shown in Table 2. Upon encountering these SPI, we also identified important virulence genes involved in fimbrial adherence (*fimA*, *C*, *D*, *F*, *H*, *I*, *W*, *Y*, *Z*), non-fimbrial adherence (*misL*), invasion (*InvA*, *B*, *C*, *E*, *F*, *G*, *H*, *I*, *J*), secretion system (*ssa*, *ssc*, *sse*, and *ssr*), magnesium uptake (*mgtB* and *mgtC*), regulation (*phoP*, *phoQ*, and *pipB*), and translocated effector (*sopB/sigD* and *sopE2*) (Table 2).

**Table 2:** Genomic features of virulence factors of *Salmonella* Typhimurium ST19 strains isolated from clinical human samples in United States.

Strain ID	SPI-1 encode genes	SPI-2 encode genes*	SPI-3 encode genes	SPI-5 encode genes	Virulence plasmid*	Fimbrial adherence determinants	SPI*
HS5302	*inv (A*, *B*, *C*, *E*, *F*, *G*, *H*, *I*, *J); sopE2*	*ssa (C*, *D*, *E*, *G*, *H*, ***I***, *J*, *K*, *L*, *M*, *N*, *O*, *P*, *Q*, *R*, *T*, *U*, *V); ssc (A*, *B); sse (B*, *C*, *D*, *E); ssr (A*, *B)*	*mgtB*, *mgtC*, *misL*	*phoP*, *phoQ*, *pipB*, *sopB/sigD*	*spv (A*, *B*, *D*, *R)*	*fim (A*, *C*, *D*, *F*, *H*, *I*, *W*, *Y*, *Z)*	SPI-1, SPI-2, SPI-3, SPI-5, SPI-13, SPI-14, C63PI
HS5344	*inv (A*, *B*, *C*, *E*, *F*, *G*, *H*, *I*, *J); sopE2*	*ssa (C*, *D*, *E*, *G*, *H*, *J*, *K*, *L*, *M*, *N*, *O*, *P*, *Q*, *R*, *T*, *U*, *V); ssc (A*, *B); sse (B*, *C*, *D*, *E); ssr (A*, *B)*	*mgtB*, *mgtC*, *misL*	*phoP*, *phoQ*, *pipB*, *sopB/sigD*	*spv (A*, *B*, *D*, *R)*	*fim (A*, *C*, *D*, *F*, *H*, *I*, *W*, *Y*, *Z)*	SPI-1, SPI-2, SPI-3, **SPI-4**, SPI-5, SPI-13, SPI-14, C63PI
HS5368	*inv (A*, *B*, *C*, *E*, *F*, *G*, *H*, *I*, *J); sopE2*	*ssa (C*, *D*, *E*, *G*, *H*, ***I***, *J*, *K*, *L*, *M*, *N*, *O*, *P*, *Q*, *R*, *T*, *U*, *V); ssc (A*, *B); sse (B*, *C*, *D*, *E); ssr (A*, *B)*	*mgtB*, *mgtC*, *misL*	*phoP*, *phoQ*, *pipB*, *sopB/sigD*	*spv (A*, *B*, *D*, *R)*	*fim (A*, *C*, *D*, *F*, *H*, *I*, *W*, *Y*, *Z)*	SPI-1, SPI-2, SPI-3, **SPI-4**, SPI-5, SPI-13, SPI-14, C63PI
HS5437	*inv (A*, *B*, *C*, *E*, *F*, *G*, *H*, *I*, *J); sopE2*	*ssa (C*, *D*, *E*, *G*, *H*, ***I***, *J*, *K*, *L*, *M*, *N*, *O*, *P*, *Q*, *R*, *T*, *U*, *V); ssc (A*, *B); sse (B*, *C*, *D*, *E); ssr (A*, *B)*	*mgtB*, *mgtC*, *misL*	*phoP*, *phoQ*, *pipB*, *sopB/sigD*	*spv (A*, *B*, *D*, *R)*	*fim (A*, *C*, *D*, *F*, *H*, *I*, *W*, *Y*, *Z)*	SPI-1, SPI-2, SPI-3, **SPI-4**, SPI-5, SPI-13, SPI-14, C63PI
HS5478	*inv (A*, *B*, *C*, *E*, *F*, *G*, *H*, *I*, *J); sopE2*	*ssa (C*, *D*, *E*, *G*, *H*, *J*, *K*, *L*, *M*, *N*, *O*, *P*, *Q*, *R*, *T*, *U*, *V); ssc (A*, *B); sse (B*, *C*, *D*, *E); ssr (A*, *B)*	*mgtB*, *mgtC*, *misL*	*phoP*, *phoQ*, *pipB*, *sopB/sigD*	*spv (A*, *B*, *D*, *R)*	*fim (A*, *C*, *D*, *F*, *H*, *I*, *W*, *Y*, *Z)*	SPI-1, SPI-2, SPI-3, SPI-5, SPI-13, SPI-14, C63PI

*Letters highlighted in bold represents differences among strains.

**Fig 2:**
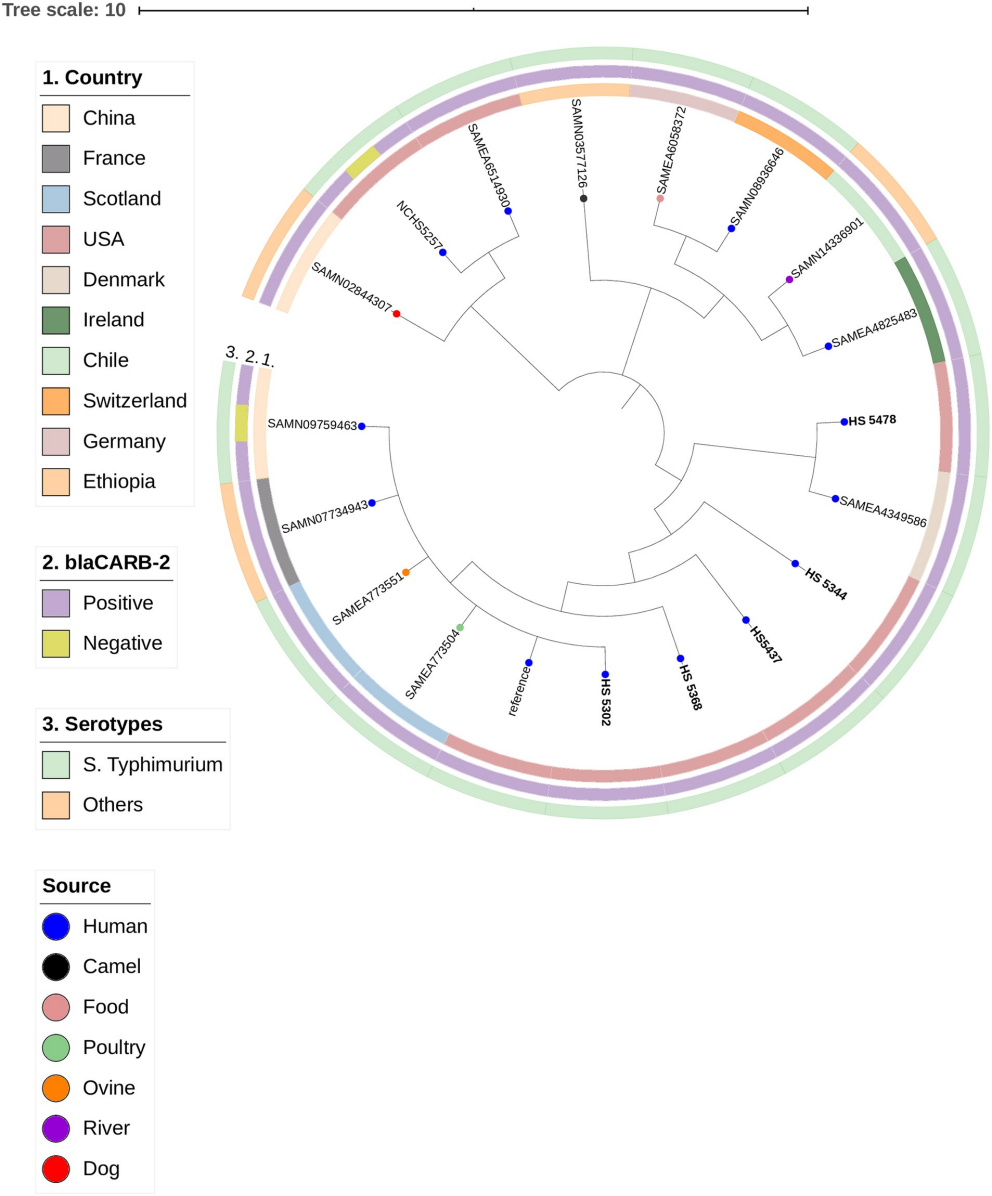
Genomic comparison between genetic contexts of virulence plasmids carried by *Salmonella* Typhimurium strains from this study (A) and *S*. *enterica* strains B (CP000858), C (NC_002638), and D (AY517905) as out-group. Genes and shotgun sequences were retrieved from the GenBank database. Arrows indicate the positions and directions of the genes; Regions with >99% identity are indicated with gray shading.

Interestingly, these strains possess a highly conserved *spv* operon composed by *spvR*, *spvA*, *spvB*, *spvC*, and *spvD* genes which are located upstream of the genes *pefA* (plasmid-encoded fimbriae) and *rck* (resistance to complement killing) in a virulence plasmid as shown in Fig 2. In addition, *in silico* analyses confirmed that these virulence genes were located on IncFIB plasmid.

**Fig 3:**
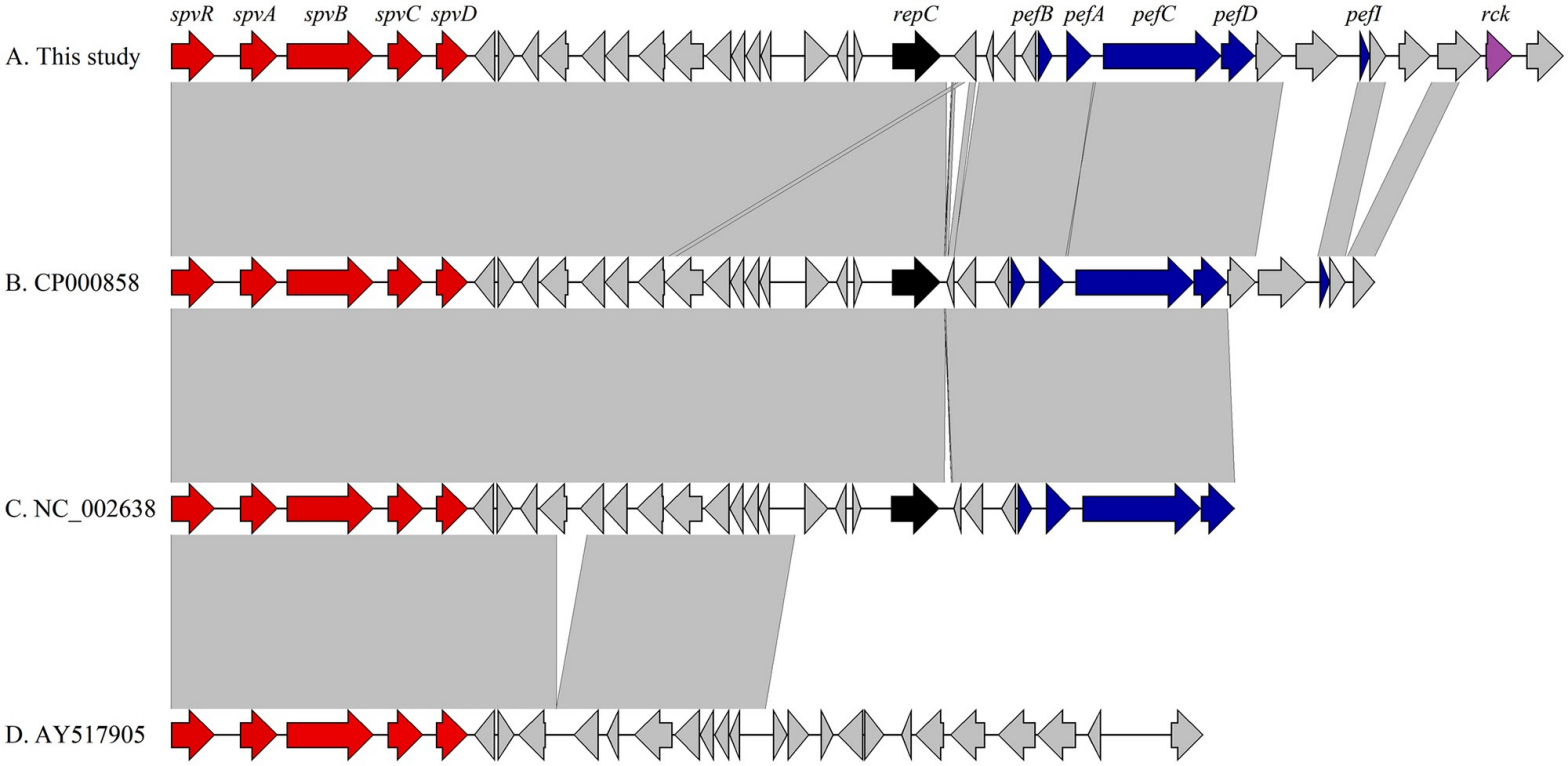
SNP-based phylogenetic tree composed by five *Salmonella* Typhimurium and additional 14 *Salmonella enterica* strains. This figure was generated with iTOL v.5.5 (https://itol.embl.de).

To achieve a better understanding of the clonal spread of these MDR strains, we reconstructed a phylogenetic tree based on SNPs. Indeed, these strains were found to be genetically related. The phylogenetic tree framed a major cluster composed by five *S*. Typhimurium strains from this study (HS5478, HS5344, HS5437, HS5302, and HS5368), which nested together with *S*. *enterica* strains from different sources (Human, poultry, ovine) and countries, including Denmark (SAMEA4349586), Scotland (SAMEA773504 and SAMEA773551), France (SAMN07734943), and China (SAMN09759463) as shown in Fig 3. Interestingly, *S*. Typhimurium strains within same cluster shared the same resistance phenotype and genotype profile.

## Discussion

The continuous dispersal of MDR *S*. *enterica* strains frequently deserves attention of the public health authorities, particularly the international lineages as *S*. Typhimurium ST19 that most often causes diseases and deaths [18, 19]. Owing to their importance, the ST19 members have been globally identified in a variety of sources, such as human clinical samples, animals, food products, and environmental samples [20–22]. Moreover, *S*. Typhimurium ST19 has shown broad resistance to a variety of critically important antimicrobials [23], including colistin (an antibiotic of last resort for some MDR infections) [24, 25]. Besides that, the occurrence of intermediate resistance reported here implies in possible treatment failure that should be noted by public health authorities.

It is important to note that these strains can easily acquire such genes through mobile genetic elements such as plasmids, integrons, and genomic islands from other MDR clones, resulting in their rapid dissemination. The presence of class 1 integron in all *S*. Typhimurium ST19 strains constitutes a risk factor to the rapid spread of antimicrobial resistance (AMR) genes. Indeed, class 1 integron coding various resistance profiles has been widely reported in *S*. Typhimurium as well as in multiple serovars [5, 21, 26–30]. This genetic frame is crucial for the spread resistance markers, since they are able to capture AMR genes through chromosomal cassettes incorporating them by site-specific recombination [4, 12, 31]. Additionally, resistance genes located in class 1 integrons are often within *Salmonella* genomic islands (SGI), such as the conjugative *Salmonella* genomic island 1 (SGI1) (~43-kb) and its variants [32, 33].

The detection of quaternary ammonium compounds (QACs) raises a particular concern, since this *qac*-containing integrons typically harbors a pool of genes that are hotspots for horizontal gene transfer providing readily adaptation to new surrounds [34, 35]. The co-resistance of critically important antimicrobials and disinfectants QACs reinforces the evidence of the overuse of biocides in clinical settings [34], and their spread have been also described in *Salmonella* serotypes isolated from livestock [36].

The *bla*_CARB-2_ gene, earlier identified as *bla*_PSE-1_, is most often a part of the chromosomal cassette [37, 38]. To date, the occurrence of this carbenicillinase gene has been limited to a few reports in different bacteria species and countries, including *Acinetobacter pittii* and *Salmonella* serovars in Australia [38, 39], *Salmonella* Typhimurium from England and Wales [40], *Salmonella* Senftenberg in Mexico [41], *S*. Typhimurium in Canada [42], *Pseudomonas aeruginosa* in Netherlands [43], and *Escherichia coli* in Pakistan [44]. It is noteworthy that such genetic element has the ability to move among different lineages of *S*. *enterica* serovars on a global scale, contributing to AMR spread [28]. Indeed, the genetic contexts surrounding *bla*_CARB-2_ gene in this study are typically found in SGI1 and its variant SGI1-B.

Drug-resistant variants of SGI1 have been identified in numerous *S*. *enterica* serovars, and strains harboring them may be more virulent and have a tendency to rapidly disseminate [33, 39]. In fact, *S*. Typhimurium strains within this survey demonstrate to possess several virulence factors, which have been reported earlier [45–48]. Furthermore, we confirmed the presence of several plasmid-borne virulence genes (*spvR*, *spvA*, *spvB*, *spvC*, *spvD*, *rck*, and *pefA*) that denotes an important genomic apparatus for the spreading of this plasmid, and may provide fitness benefit as previously reported [28, 49, 50]. Increasing evidences have demonstrated that the *spv* operon affects the formation of autophagosomes, as well as highlight its association in killing of macrophages and neutrophils [6], being crucially required for virulence *in vivo* [8], including aggravated damage in zebrafish infection model [7]. Furthermore, the PhoP-regulated gene *mig-14* that is required for virulence and resistance to antimicrobial peptides was detected in these strains. Yet, *mig-14* contributes to *Salmonella* persistence in hosts, being also associated with resistance against polymyxin B and cathelin-related antimicrobial peptide (CRAMP) [51–54]. Thus, the clonal dissemination of MDR *S*. Typhimurium (mostly the invasive clones) constitutes an important issue to public health [55], especially *S*. Typhimurium ST19, which have been circulating worldwide (http://enterobase.warwick.ac.uk/) as demonstrated in this study, since our *S*. Typhimurium strains nested with international lineages from at least four countries (Fig 2).

In summary, we report the genomic features of virulent and MDR *S*. Typhimurium ST19 strains carrying the *bla*_CARB-2_ gene recovered from clinical human samples in United States. Our results provide new insights into this genetic environment that besides *bla*_CARB-2_, contains genes, coding resistance to quaternary ammonium compounds (*qacEΔ1*) and sulfonamides (*sul1*). Furthermore, our findings could aid in understanding the epidemiology of *S*. Typhimurium ST19, which are of great value to initiate preventive measures to safeguard human health. Given the high spread of this international lineage, especially among the young and the elderly or immunocompromised people, public health authorities and regulatory food agencies need to be aware of the potential impact in public health and in economy caused by such pandemic MDR *S*. Typhimurium ST19 lineage, with particular attention in high-burden areas.

## References

[1] Campos J, Mourão J, Peixe L, Antunes P. Non-typhoidal *Salmonella* in the Pig Production Chain: A Comprehensive Analysis of Its Impact on Human Health. Pathogens. 2019; 8(1):E19 10.3390/pathogens8010019. 30700039 PMC6470815

[2] Almeida F, Seribelli AA, Medeiros MIC, Rodrigues DDP, de Mello Varani A, Luo Y, et al Phylogenetic and antimicrobial resistance gene analysis of *Salmonella* Typhimurium strains isolated in Brazil by whole genome sequencing. PLoS One. 2018; 13(8):e0201882 10.1371/journal.pone.0201882. 30102733 PMC6089434

[3] Monte DF, Lincopan N, Fedorka-Cray P, Landgraf M. Current Insights on High Priority Antibiotic-Resistant *Salmonella enterica* in Food and Foodstuffs: A review. Curr Opin Food Sci. 2019; 26:35–46. 10.1016/j.cofs.2019.03.004.

[4] Ghaly TM, Chow L, Asher AJ, Waldron LS, Gillings MR. Evolution of class 1 integrons: Mobilization and dispersal via food-borne bacteria. PLoS One. 2017; 12(6):e0179169 10.1371/journal.pone.0179169. 28586403 PMC5460862

[5] Pornsukarom S, Thakur S. Horizontal Dissemination of Antimicrobial Resistance Determinants in Multiple *Salmonella* Serotypes following Isolation from the Commercial Swine Operation Environment after Manure Application. Appl Environ Microbiol. 2017; 83(20):e01503–17. 10.1128/AEM.01503-17. 28802274 PMC5626988

[6] Wu SY, Wang LD, Li JL, Xu GM, He ML, Li YY, et al *Salmonella spv* locus suppresses host innate immune responses to bacterial infection. Fish Shellfish Immunol. 2016; 58:387–396. 10.1016/j.fsi.2016.09.042. 27666190

[7] Wu SY, Wang LD, Xu GM, Yang SD, Deng QF, Li YY, et al *spv* locus aggravates *Salmonella* infection of zebrafish adult by inducing Th1/Th2 shift to Th2 polarization. Fish Shellfish Immunol. 2017; 67:684–691. 10.1016/j.fsi.2017.06.057. 28666864

[8] Passaris I, Cambré A, Govers SK, Aertsen A. Bimodal Expression of the *Salmonella* Typhimurium *spv* Operon. Genetics. 2018; 210(2):621–635. 10.1534/genetics.118.300822. 30143595 PMC6216589

[9] Clinical and Laboratory Standards Institute. 2019. Performance standards for antimicrobial susceptibility testing. 29th ed. CLSI supplement M100. Clinical and Laboratory Standards Institute, Wayne, PA.

[10] Clinical Laboratory Standards Institute. 2015. Performance Standards for Antimicrobial Disk and Dilution Susceptibility Tests for Bacteria Isolated From Animals. 3rd ed. CLSI Supplement VET01S. CLSI, Wayne, PA.

[11] Magiorakos AP, Srinivasan A, Carey RB, Carmeli Y, Falagas ME, Giske CG, et al Multidrug-resistant, extensively drug-resistant and pandrug-resistant bacteria: an international expert proposal for interim standard definitions for acquired resistance. Clin Microbiol Infect. 2012; 18(3):268–81. 10.1111/j.1469-0691.2011.03570.x. 21793988

[12] Gebreyes WA, Thakur S, Davies PR, Funk JA, Altier C. Trends in antimicrobial resistance, phage types and integrons among *Salmonella* serotypes from pigs, 1997–2000. J Antimicrob Chemother. 2004; 53(6):997–1003. 10.1093/jac/dkh247. 15150175

[13] Rao S, Maddox CW, Hoien-Dalen P, Lanka S, Weigel RM. Diagnostic accuracy of class 1 integron PCR method in detection of antibiotic resistance in *Salmonella* isolates from swine production systems. J Clin Microbiol. 2008; 46(3):916–20. 10.1128/JCM.01597-07. 18174294 PMC2268369

[14] Pornsukarom S, van Vliet AHM, Thakur S. Whole genome sequencing analysis of multiple *Salmonella* serovars provides insights into phylogenetic relatedness, antimicrobial resistance, and virulence markers across humans, food animals and agriculture environmental sources. BMC Genomics. 2018; 19(1):801 10.1186/s12864-018-5137-4. 30400810 PMC6218967

[15] Monte DF, Nelson V, Cerdeira L, Keelara S, Greene S, Griffin D, et al Multidrug- and colistin-resistant *Salmonella enterica* 4,[5],12:i:- sequence type 34 carrying the mcr-3.1 gene on the IncHI2 plasmid recovered from a human. J Med Microbiol. 2019; 68(7):986–990. 10.1099/jmm.0.001012. 31162025 PMC6939160

[16] Liu B, Zheng D, Jin Q, Chen L, Yang J. VFDB 2019: a comparative pathogenomic platform with an interactive web interface. Nucleic Acids Res. 2019; 47(D1):D687–D692. 10.1093/nar/gky1080. 30395255 PMC6324032

[17] Kaas RS, Leekitcharoenphon P, Aarestrup FM, Lund O. Solving the problem of comparing whole bacterial genomes across different sequencing platforms. PLoS One. 2014; 9(8):e104984 10.1371/journal.pone.0104984. 25110940 PMC4128722

[18] World Health Organization. 2015. WHO estimates of the global burden of foodborne diseases, 2007–2015 WHO, Geneva, Switzerland.

[19] Carroll LM, Wiedmann M, den Bakker H, Siler J, Warchocki S, Kent D, et al Whole-Genome Sequencing of Drug-Resistant *Salmonella enterica* Isolates from Dairy Cattle and Humans in New York and Washington States Reveals Source and Geographic Associations. Appl Environ Microbiol. 2017; 83(12): e00140–17. 10.1128/AEM.00140-17. 28389536 PMC5452826

[20] Gymoese P, Sørensen G, Litrup E, Olsen JE, Nielsen EM, Torpdahl M. Investigation of Outbreaks of *Salmonella enterica* Serovar Typhimurium and Its Monophasic Variants Using Whole-Genome Sequencing, Denmark. Emerg Infect Dis. 2017; 23(10):1631–1639. 10.3201/eid2310.161248. 28930002 PMC5621559

[21] Jain P, Sudhanthirakodi S, Chowdhury G, Joshi S, Anandan S, Ray U, et al Antimicrobial resistance, plasmid, virulence, multilocus sequence typing and pulsed-field gel electrophoresis profiles of *Salmonella enterica* serovar Typhimurium clinical and environmental isolates from India. PLoS One. 2018; 13(12):e0207954 10.1371/journal.pone.0207954. 30540810 PMC6291080

[22] Panzenhagen PHN, Paul NC, Conte CA Jr, Costa RG, Rodrigues DP, Shah DH. Genetically distinct lineages of *Salmonella* Typhimurium ST313 and ST19 are present in Brazil. Int J Med Microbiol. 2018; 308(2):306–316. 10.1016/j.ijmm.2018.01.005. 29396155

[23] Critically important antimicrobials for human medicine– 5th rev. Geneva: World Health Organization. 2017; License: CC BY-NC-SA 3.0 IGO.

[24] Rau RB, de Lima-Morales D, Wink PL, Ribeiro AR, Martins AF, Barth AL. Emergence of *mcr-1* Producing *Salmonella enterica* serovar Typhimurium from Retail Meat: First Detection in Brazil. Foodborne Pathog Dis. 2018; 15(1):58–59. 10.1089/fpd.2017.2346. 29048947

[25] Rau RB, de Lima-Morales D, Wink PL, Ribeiro AR, Barth AL. *Salmonella enterica mcr-1* Positive from Food in Brazil: Detection and Characterization. Foodborne Pathog Dis. 2020; 17(3):202–208. 10.1089/fpd.2019.2700. 31556704

[26] Keelara S, Scott HM, Morrow WM, Hartley CS, Griffin DL, Gebreyes WA, et al Comparative phenotypic and genotypic characterization of temporally related nontyphoidal *Salmonella* isolated from human clinical cases, pigs, and the environment in North Carolina. Foodborne Pathog Dis. 2014; 11(2):156–64. 10.1089/fpd.2013.1630. 24237109

[27] Ahmed HA, El-Hofy FI, Shafik SM, Abdelrahman MA, Elsaid GA. Characterization of virulence-associated genes, antimicrobial resistance genes and Class 1 integrons in *Salmonella enterica* serovar Typhimurium isolates from chicken meat and humans in Egypt. Foodborne Pathog Dis. 2016; 13:281–288. 10.1089/fpd.2015.2097. 26977940

[28] Lopes GV, Michael GB, Cardoso M, Schwarz S. Antimicrobial resistance and class 1 integron-associated gene cassettes in *Salmonella enterica* serovar Typhimurium isolated from pigs at slaughter and abattoir environment. Vet Microbiol. 2016; 194:84–92. 10.1016/j.vetmic.2016.04.020. 27142182

[29] Yang J, Ju Z, Yang Y, Zhao X, Jiang Z, Sun S. Serotype, antimicrobial susceptibility and genotype profiles of *Salmonella* isolated from duck farms and a slaughterhouse in Shandong province, China. BMC Microbiol. 2019; 19(1):202 10.1186/s12866-019-1570-z. 31477003 PMC6720067

[30] Siriken B, Al G, Erol I. Prevalence and Antibiotic Resistance of *Salmonella* Enteritidis and *Salmonella* Typhimurium in Ground Beef and Meatball Samples in Samsun, Turkey. Microb Drug Resist. 2020; 26(2):136–144. 10.1089/mdr.2018.0481. 31453743

[31] Argüello H, Guerra B, Rodríguez I, Rubio P, Carvajal A. Characterization of Antimicrobial Resistance Determinants and Class 1 and Class 2 Integrons in *Salmonella enterica* spp., Multidrug-Resistant Isolates from Pigs. Genes (Basel). 2018; 9(5): E256 10.3390/genes9050256. 29772742 PMC5977196

[32] Doublet B, Boyd D, Mulvey MR, Cloeckaert A. The *Salmonella* genomic island 1 is an integrative mobilizable element. Mol Microbiol. 2005; 55(6):1911–24. 10.1111/j.1365-2958.2005.04520.x. 15752209

[33] Mulvey MR, Boyd DA, Olson AB, Doublet B, Cloeckaert A. The genetics of *Salmonella* genomic island 1. Microbes Infect. 2006; 8(7):1915–22. 10.1016/j.micinf.2005.12.028. 16713724

[34] Hegstad K, Langsrud S, Lunestad BT, Scheie AA, Sunde M, Yazdankhah SP. Does the wide use of quaternary ammonium compounds enhance the selection and spread of antimicrobial resistance and thus threaten our health? Microb Drug Resist. 2010; 16(2):91–104. 10.1089/mdr.2009.0120. 20370507

[35] Rajamohan G, Srinivasan VB, Gebreyes WA. Biocide-tolerant multidrug-resistant *Acinetobacter baumannii* clinical strains are associated with higher biofilm formation. J Hosp Infect. 2009; 73(3):287–289. 10.1016/j.jhin.2009.07.015. 19762119

[36] de Quadros CL, Manto L, Mistura E, Webber B, Ritterbusch GA, Borges KA, et al Antimicrobial and Disinfectant Susceptibility of *Salmonella* Serotypes Isolated from Swine Slaughterhouses. Curr Microbiol. 2020; 77(6):1035–1042. 10.1007/s00284-020-01904-9. 32008078

[37] Huovinen P, Jacoby GA. Sequence of the PSE-1 beta-lactamase gene. Antimicrob Agents Chemother. 1991; 35:2428–2430. 10.1128/aac.35.11.2428. 1804019 PMC245398

[38] Kamolvit W, Derrington P, Paterson DL, Sidjabat HE. A case of IMP-4-, OXA-421-, OXA-96-, and CARB-2-producing *Acinetobacter pittii* sequence type 119 in Australia. J Clin Microbiol. 2015; 53(2):727–30. 10.1128/JCM.02726-14. 25428154 PMC4298557

[39] Levings RS, Lightfoot D, Partridge SR, Hall RM, Djordjevic SP. The genomic island SGI1, containing the multiple antibiotic resistance region of *Salmonella enterica* serovar Typhimurium DT104 or variants of it, is widely distributed in other S. enterica serovars. J Bacteriol. 2005; 187(13):4401–9. 10.1128/JB.187.13.4401-4409.2005. 15968049 PMC1151792

[40] Walker RA, Lindsay E, Woodward MJ, Ward LR, Threlfall EJ. Variation in clonality and antibiotic-resistance genes among multiresistant *Salmonella enterica* serotype Typhimurium phage-type U302 (MR U302) from humans, animals, and foods. Microb Drug Resist. 2001; 7(1):13–21. 10.1089/107662901750152701. 11310799

[41] Delgado-Suárez EJ, Ortíz-López R, Gebreyes WA, Allard MW, Barona-Gómez F, Rubio-Lozano MS. Genomic surveillance links livestock production with the emergence and spread of multi-drug resistant non-typhoidal *Salmonella* in Mexico. J Microbiol. 2019; 57(4):271–280. 10.1007/s12275-019-8421-3. 30721457

[42] Ng LK, Mulvey MR, Martin I, Peters GA, Johnson W. Genetic characterization of antimicrobial resistance in Canadian isolates of *Salmonella* serovar Typhimurium DT104. Antimicrob Agents Chemother. 1999; 43(12):3018–21. 10.1128/AAC.43.12.3018. 10582902 PMC89607

[43] van der Zee A, Kraak WB, Burggraaf A, Goessens WHF, Pirovano W, Ossewaarde JM, et al Spread of Carbapenem Resistance by Transposition and Conjugation Among *Pseudomonas aeruginosa*. Front Microbiol. 2018; 9:2057 10.3389/fmicb.2018.02057. 30233535 PMC6133989

[44] Mohsin M, Azam M, Ur Rahman S, Esposito F, Sellera FP, Monte DF, et al Genomic background of a colistin-resistant and highly virulent MCR-1-positive *Escherichia coli* ST6395 from a broiler chicken in Pakistan. Pathog Dis. 2019; 77(7):ftz064 10.1093/femspd/ftz064. 31782775

[45] Marcus SL, Brumell JH, Pfeifer CG, Finlay BB. *Salmonella* pathogenicity islands: big virulence in small packages. Microbes Infect. 2000; 2:145–56, 10.1016/S1286-4579(00)00273-2. 10742687

[46] Tang T, Cheng A, Wang M, Li X. Reviews in *Salmonella* Typhimurium PhoP/PhoQ two-component regulatory system. Rev Med Microbiol. 2013; 24:18–21, 10.1097/MRM.0b013e32835a9490.

[47] Abd El Ghany M, Shi X, Li Y, Ansari HR, Hill-Cawthorne GA, Ho YS, et al Genomic and Phenotypic Analyses Reveal the Emergence of an Atypical *Salmonella enterica* Serovar Senftenberg Variant in China. J Clin Microbiol. 2016; 54(8):2014–22. 10.1128/JCM.00052-16. 27225410 PMC4963490

[48] Monte DF, Lincopan N, Berman H, Cerdeira L, Keelara S, Thakur S, et al Genomic Features of High-Priority *Salmonella enterica* Serovars Circulating in the Food Production Chain, Brazil, 2000–2016. Sci Rep. 2019; 9(1):11058 10.1038/s41598-019-45838-0. 31363103 PMC6667439

[49] Guerra B, Soto S, Helmuth R, Mendoza MC. Characterization of a self-transferable plasmid from *Salmonella enterica* serotype Typhimurium clinical isolates carrying two integron-borne gene cassettes together with virulence and drug resistance genes. Antimicrob Agents Chemother. 2002; 46(9):2977–81. 10.1128/aac.46.9.2977-2981.2002. 12183256 PMC127424

[50] Carattoli A. Resistance plasmid families in Enterobacteriaceae. Antimicrob Agents Chemother. 2009; 53(6):2227–38. 10.1128/AAC.01707-08. 19307361 PMC2687249

[51] Navarre WW, Halsey TA, Walthers D, Frye J, McClelland M, Potter JL, et al Co-regulation of *Salmonella enterica* genes required for virulence and resistance to antimicrobial peptides by SlyA and PhoP/PhoQ. Mol Microbiol. 2005; 56(2):492–508. 10.1111/j.1365-2958.2005.04553.x. 15813739

[52] Brodsky IE, Ghori N, Falkow S, Monack D. Mig-14 is an inner membrane-associated protein that promotes *Salmonella* Typhimurium resistance to CRAMP, survival within activated macrophages and persistent infection. Mol Microbiol. 2005; 55(3):954–72. 10.1111/j.1365-2958.2004.04444.x. 15661016

[53] Brodsky IE, Ernst RK, Miller SI, Falkow S. mig-14 is a *Salmonella* gene that plays a role in bacterial resistance to antimicrobial peptides. J Bacteriol. 2002; 184(12):3203–13. 10.1128/jb.184.12.3203-3213.2002. 12029036 PMC135090

[54] Sheng X, Wang W, Chen L, et al Mig-14 may contribute to *Salmonella enterica* serovar Typhi resistance to polymyxin B by decreasing the permeability of the outer-membrane and promoting the formation of biofilm. Int J Med Microbiol. 2019; 309(2):143–150. 10.1016/j.ijmm.2019.01.001. 30685290

[55] GBD 2017 Non-Typhoidal *Salmonella* Invasive Disease Collaborators. The global burden of non-typhoidal *Salmonella* invasive disease: a systematic analysis for the Global Burden of Disease Study 2017. Lancet Infect Dis. 2019; 19(12):1312–1324. 10.1016/S1473-3099(19)30418-9. 31562022 PMC6892270

