# Correction: Class 1 integron-borne cassettes harboring *bla*_CARB-2_ gene in multidrug-resistant and virulent *Salmonella* Typhimurium ST19 strains recovered from clinical human stool samples, United States

**DOI:** 10.1371/journal.pone.0249536

**Published:** 2021-03-29

**Authors:** Daniel F. M. Monte, Fábio P. Sellera, Ralf Lopes, Shivaramu Keelara, Mariza Landgraf, Shermalyn Greene, Paula J. Fedorka-Cray, Siddhartha Thakur

The images for Figs 2 and 3 are incorrectly switched. The image that appears as Fig 2 should be Fig 3, and the image that appears as Fig 3 should be Fig 2. The figure captions appear in the correct order.

**Fig 2:**
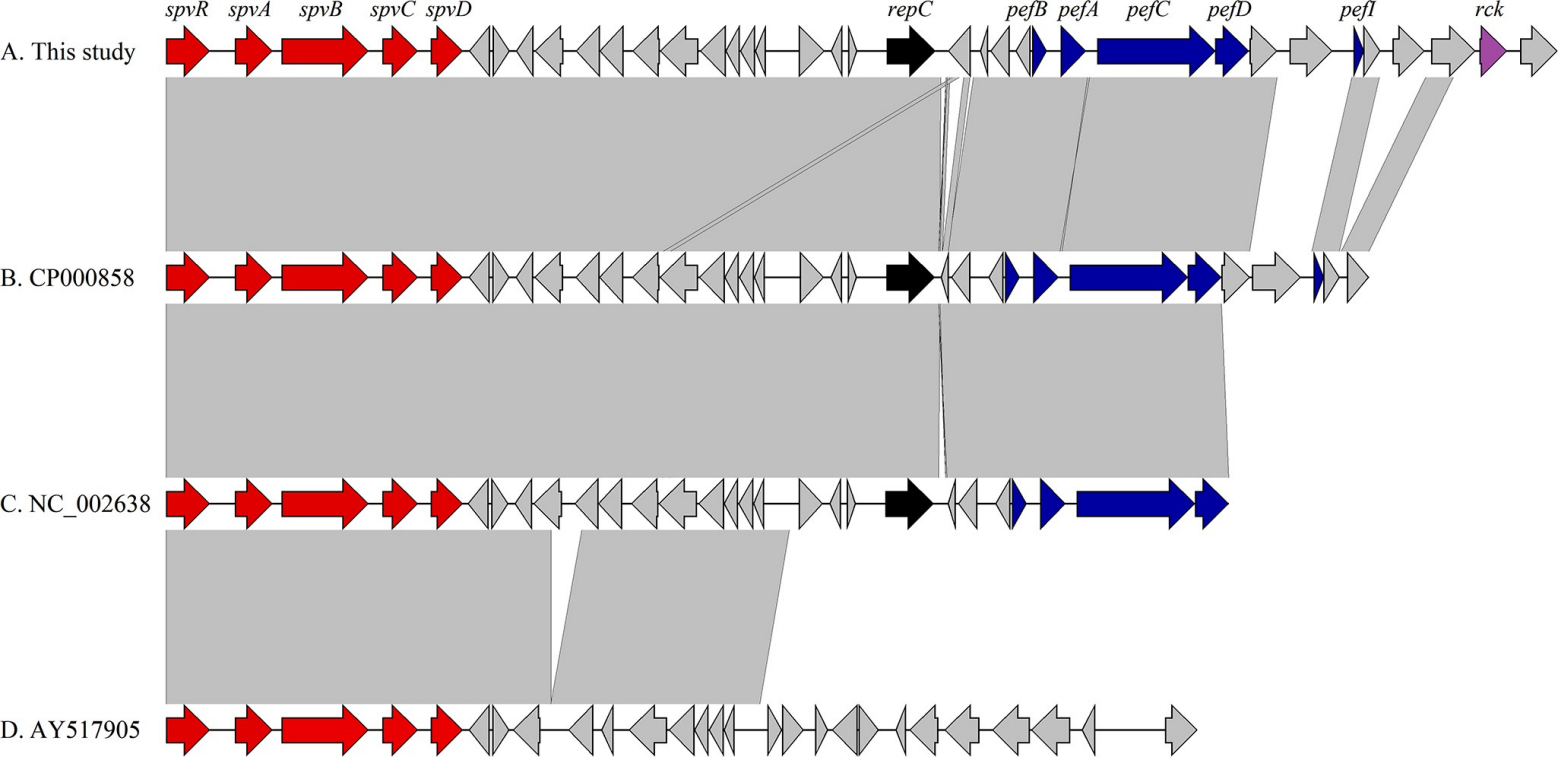
Genomic comparison between genetic contexts of virulence plasmids carried by *Salmonella* Typhimurium strains from this study (A) and *S*. *enterica* strains B (CP000858), C (NC_002638), and D (AY517905) as out-group. Genes and shotgun sequences were retrieved from the GenBank database. Arrows indicate the positions and directions of the genes; Regions with >99% identity are indicated with gray shading.

**Fig 3:**
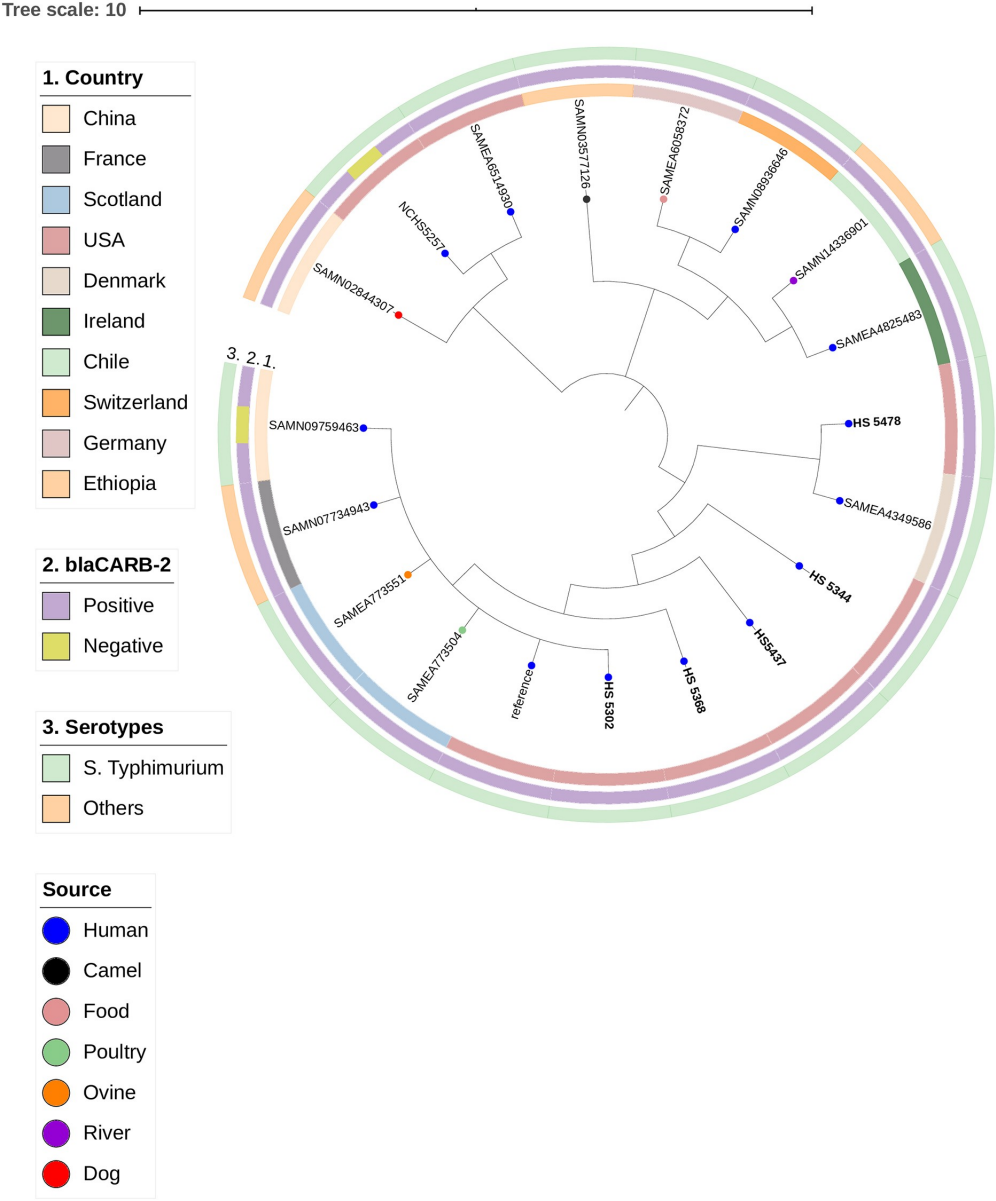
SNP-based phylogenetic tree composed by five *Salmonella* Typhimurium and additional 14 *Salmonella enterica* strains. This figure was generated with iTOL v.5.5 (https://itol.embl.de).
