# Supplementary material for: Class 1 integron-borne cassettes harboring blaCARB-2 gene in multidrug-resistant and virulent Salmonella Typhimurium ST19 strains recovered from clinical human stool samples, United States
Source: PLoS One. 2020 Oct 30;15(10):e0240978. doi: 10.1371/journal.pone.0240978 (PMC7598458; doi:10.1371/journal.pone.0240978)
Supplement: Attachment — Submitted filename: Salmonella ST19.docx [file pone.0240978.s001.docx]

This manuscript describes about the class 1 integron AMR cassettes harboring *bla*_CARB-2_ gene in MDR *Salmonella* Typhimurium ST19 isolates. These MDR isolates are also harbored several virulence encoding genes and SPIs.

Comments:

1. Based on the WHO’s description, some of the antimicrobial such as ticarcillin/clavulanic acid and tetracycline are not critically and highly important  antimicrobials.
2. Lines 69-70. ‘CARB-2-production’ was not tested in these S. Typhimurium isolates, but only the encoding gene was detected.
3. Line 75. It will be useful if the nature of clinical source (watery diarrhea, diarrhea with mucus or dysentery-like stool) of the isolates and clinical history of the patients described and included. In addition, the year/location of isolation is also important to be mentioned in the text to deduce whether the isolates are spread all over the country or restricted to a certain geographical location.
4. Lines 100-101. What is the rational for selecting only 13 sequences for comparison? There other STs (e.g., ST39) that expresses more AMR than the ST19. It will be interesting to consider all the MDR S. Typhimurium STs from different sources.
5. Lines 114-115. It is not surprising, as these intermediate  resistant isolates to doxycycline are also resistant to tetracycline. Doxycycline is a synthetic antibiotic derived from tetracycline.
6. Lines 146-151. It will be interesting to compare these putative virulence encoding genes and SPIs with the infection status of the patients (pl refer comment #3).
7. The title of the manuscript reflects only the MDR part, but not about the virulence.
8. There is inconsistency while using the terms isolate and strain.
9. Only the *bla*_CARB-2_  was mapped in the class 1 integron. Are the other genes (*aac(6’)-Iaa, aadA2b, aph(3’)-Ia,* *sul1, tetG, floR,*) are located in the same AMR cassettes in the integron? How they are arranged on the chromosome?

Review Comments to the Author

**Minimum Character Count Not Met**

Dear Editorial Team,

I am unable to submit my comments, as the system shows “Minimum Character count not met” in the Review comments to the Author. The ward count of my comments is 1929
