# Supplementary material for: Class 1 integron-borne cassettes harboring blaCARB-2 gene in multidrug-resistant and virulent Salmonella Typhimurium ST19 strains recovered from clinical human stool samples, United States
Source: PLoS One. 2020 Oct 30;15(10):e0240978. doi: 10.1371/journal.pone.0240978 (PMC7598458; doi:10.1371/journal.pone.0240978)
Supplement: Attachment — Submitted filename: R1_Response to reviewers_PONE-D-20-21578_DM-Sid.docx [file pone.0240978.s002.docx]

**Response to reviewers, Manuscript PONE-D-20-21578.R1**

Line numbers in our response refer to the line numbers in the revised manuscript (marked-up).

**General comments:**

**We sincerely appreciate the editorial board and reviewers for their careful review and constructive suggestions. It is our belief that the manuscript was substantially improved after making the suggested edits. We have responded to specific queries below.**

Comments from Reviewers:

Reviewer #1:

This manuscript describes about the class 1 integron AMR cassettes harboring *bla*_CARB-2_ gene in MDR *Salmonella* Typhimurium ST19 isolates. These MDR isolates are also harbored several virulence encoding genes and SPIs.

**Response: We sincerely thank the reviewer for the favorable comments.**

Comments:

Based on the WHO’s description, some of the antimicrobial such as ticarcillin/clavulanic acid and tetracycline are not critically and highly important antimicrobials.

**Response: We appreciate your suggestion. However, based on this document: Critically important antimicrobials for human medicine, 6th revision. Geneva: World Health Organization; 2019.** <https://apps.who.int/iris/bitstream/handle/10665/312266/9789241515528-eng.pdf?sequence=1&isAllowed=y>, **we could observe that ticarcillin/clavulanic acid and tetracycline are classified as critically and highly important antimicrobials.**

Lines 69-70. ‘CARB-2-production’ was not tested in these *S*. Typhimurium isolates, but only the encoding gene was detected.

**Response: We agree. This has been deleted in lines 72 and 252.**

Line 75. It will be useful if the nature of clinical source (watery diarrhea, diarrhea with mucus or dysentery-like stool) of the isolates and clinical history of the patients described and included. In addition, the year/location of isolation is also important to be mentioned in the text to deduce whether the isolates are spread all over the country or restricted to a certain geographical location.

**Response: We fully agree. At the beginning of this study, we tried to collect such information to conduct the paper describing the clinical history of the patients. In our understanding, such information could aid to mitigate strategies, as well as give to readers a full background, which denotes the real threat of salmonellosis and their impacts in human health. However, we did not get permission from Public Health Laboratory due to health information privacy to publish this information. Therefore, we respectfully request that the paper be published with the information provided.**

Lines 100-101. What is the rational for selecting only 13 sequences for comparison? There other STs (e.g., ST39) that expresses more AMR than the ST19. It will be interesting to consider all the MDR *S*. Typhimurium STs from different sources.

**Response: Our intention was to cover several countries (n=10) and sources (n=7) in this phylogeny by using *S. enterica* strains harboring *bla*_CARB-2_ gene, with exception of two strains that were used as out-groups.**

Lines 114-115. It is not surprising, as these intermediate  resistant isolates to doxycycline are also resistant to tetracycline. Doxycycline is a synthetic antibiotic derived from tetracycline.

**Response: We agree. This has been modified in lines 124-126.**

**“Moreover, intermediate resistance to doxycycline was detected in three strains (HS5344, HS5437 and HS5478), and in a single strain (HS5437) to ceftazidime”.**

Lines 146-151. It will be interesting to compare these putative virulence encoding genes and SPIs with the infection status of the patients (pl refer comment #3).

**Response: We agree. However, as prior mentioned, we are unable to provide this information.**

The title of the manuscript reflects only the MDR part, but not about the virulence.

**Response: We agree. The title has been modified. “Class 1 Integron-Borne Cassettes harboring *bla*_CARB-2_ gene in Multidrug-Resistant and Virulent *Salmonella* Typhimurium ST19 Strains recovered from clinical human stool samples, United States”. (Lines 2-4).**

There is inconsistency while using the terms isolate and strain.

**Response: Thank you for this observation. Since the main target of this study was the genomic investigation and all *Salmonella* Typhimurium were accessed from a collection, we believe that the term strain is the most appropriate. Therefore, we have replaced these terms throughout the manuscript in lines 31, 33, 72, 83, 84, 85, 90, 100, 121, 125, 126, 127, 133, 170, 183, 244, and 252.**

Only the *bla*_CARB-2_  was mapped in the class 1 integron. Are the other genes (*aac(6’)-Iaa, aadA2b, aph(3’)-Ia,* *sul1, tetG, floR,*) are located in the same AMR cassettes in the integron? How they are arranged on the chromosome?

**Response: This information was added to the revised version of the manuscript. Lines 144-154: “****Additionally, the *sul1*, *bla*_CARB-2_, *tetG*, *floR*, and *aadA2b* resistance genes were harbored by a partial sequence of a complex class 1 integron (In104) from HS5344, HS5437, and HS5478. This sequence included duplications of parts of the integron conserved segments (CS), specifically, part of the *intI1* gene from the 5’-CS and part of the 3’-CS (*qacE∆1* and partial *sul1* genes). Consequently, the structure had two attI1 sites, into which the *aadA2b* gene cassette was inserted in one and the *bla*_CARB-2_ cassette in the other. The *floR* and *tetG* genes were identified between the two integron-derived regions. In HS5302 and HS5368, only the region containing the *intI1*-*bla*_CARB-2_-*qacE∆1*-*sul1*-orf5-orf6 array was detected. Furthermore, while *aac(6’)-Iaa* was found at a site distant from the other resistance genes on the chromosome of all *S*. Typhimurium strains in this study, *aph(3’)-Ia* was identified in a partial transposon sequence from HS5478.”**

**Additional changes:**

**The following sentence has been added to material and methods as per standard protocol for publications in PLoS One.**

**“Ethics approval and consent to participate**

**The human patients from whom *Salmonella* were recovered were completely anonymous and even after all the analysis and tests, the human sample remained anonymous. As such, the NC State IRB (FWA: 00003429) indicated the study research did not need IRB approval because it does not meet the definition for human subjects research”. (Lines 77-81).**
